# Influence of ligand’s directional configuration, chrysenes as model compounds, on the binding activity with aryl hydrocarbon receptor

**DOI:** 10.1038/s41598-020-70704-9

**Published:** 2020-08-14

**Authors:** Taewoo Kim, Juyuan Zhen, Junghyun Lee, Robert Bauer, Changkeun Lee, Bong-Oh Kwon, Keun Hwa Chae, Seongjin Hong, John P. Giesy, Gap Soo Chang, Jong Seong Khim

**Affiliations:** 1 School of Earth and Environmental Sciences and Research Institute of Oceanography, Seoul National University, Seoul, Republic of Korea; 2 Department of Physics and Engineering Physics, University of Saskatchewan, Saskatoon, SK Canada; 3 Department of Marine Biotechnology, Kunsan National University, Kunsan, Republic of Korea; 4 Advanced Analysis Center, Korea Institute of Science and Technology, Seoul, Republic of Korea; 5 Department of Ocean Environmental Sciences, Chungnam National University, Daejeon, Republic of Korea; 6 Department of Veterinary Biomedical Sciences and Toxicology Centre, University of Saskatchewan, Saskatoon, SK Canada; 7 Environmental Sciences Department, Baylor University, Waco, TX USA

**Keywords:** Computational chemistry, Density functional theory, Computational biophysics

## Abstract

Understanding what and how physico-chemical factors of a ligand configure conditions for ligand-receptor binding is a key to accurate assessment of toxic potencies of environmental pollutants. We investigated influences of the dipole-driven orientation and resulting directional configuration of ligands on receptor binding activities. Using physico-chemical properties calculated by ab initio density functional theory, directional reactivity factors (DRF) were devised as main indicators of toxic potencies, linking molecular ligand-receptor binding to in vitro responses. The directional reactive model was applied to predict variation of aryl hydrocarbon receptor-mediated toxic potencies among homologues of chrysene with structural modifications such as the numbers of constituent benzene rings, methylation and hydroxylation. Results of predictive models were consistent with empirical potencies determined by use of the H4IIE-*luc* transactivation bioassay. The experiment-free approach based on first principles provides an analytical framework for estimating molecular bioactivity in silico and complements conventional empirical approaches to studying molecular initiating events in adverse outcome pathways.

## Introduction

Protein receptors act as transcription factors that regulate expressions of genes and determine how cells in organisms respond to xenobiotics. When a receptor binds with exogenous substances (viz., ligand), ligand-receptor complexes are translocated to nuclei of cells where they regulate expressions of genes. In the case of xenobiotics, this includes up-regulation of genes coding for enzymes, such as cytochrome P450 1A1 (CYP1A1), which transforms xenobiotics^1^. This receptor-mediated, enzyme regulation depends on ligand-specific binding affinity and especially since it is the first initiation event, provides the basis of toxicology. Although various receptors and signal transduction pathways are known, kinetics of how ligands interact with responsive receptors remains imperfect and is thus currently undergoing intensive research^2,3^.

To understand such ligand-specific events involved in adverse outcome pathways (AOPs) or functioning of drugs, knowledge of affinities of binding to receptors is fundamental^4^. There are a number of nuclear receptors, conserved in a wide range of vertebrates with which contaminants or drugs can interact as agonists or antagonists^5^. Variations among amino acid sequences of the ligand binding domain can explain differences in potencies of chemicals and sensitivities among species^6^. Due to incomplete understanding of physico-chemical interactions involved in binding of ligands to protein receptors, accurate prediction of affinities of ligand-specific binding in addition to steric factors, predicting initiation of AOPs, and toxicities is challenging.

**Figure 1:**
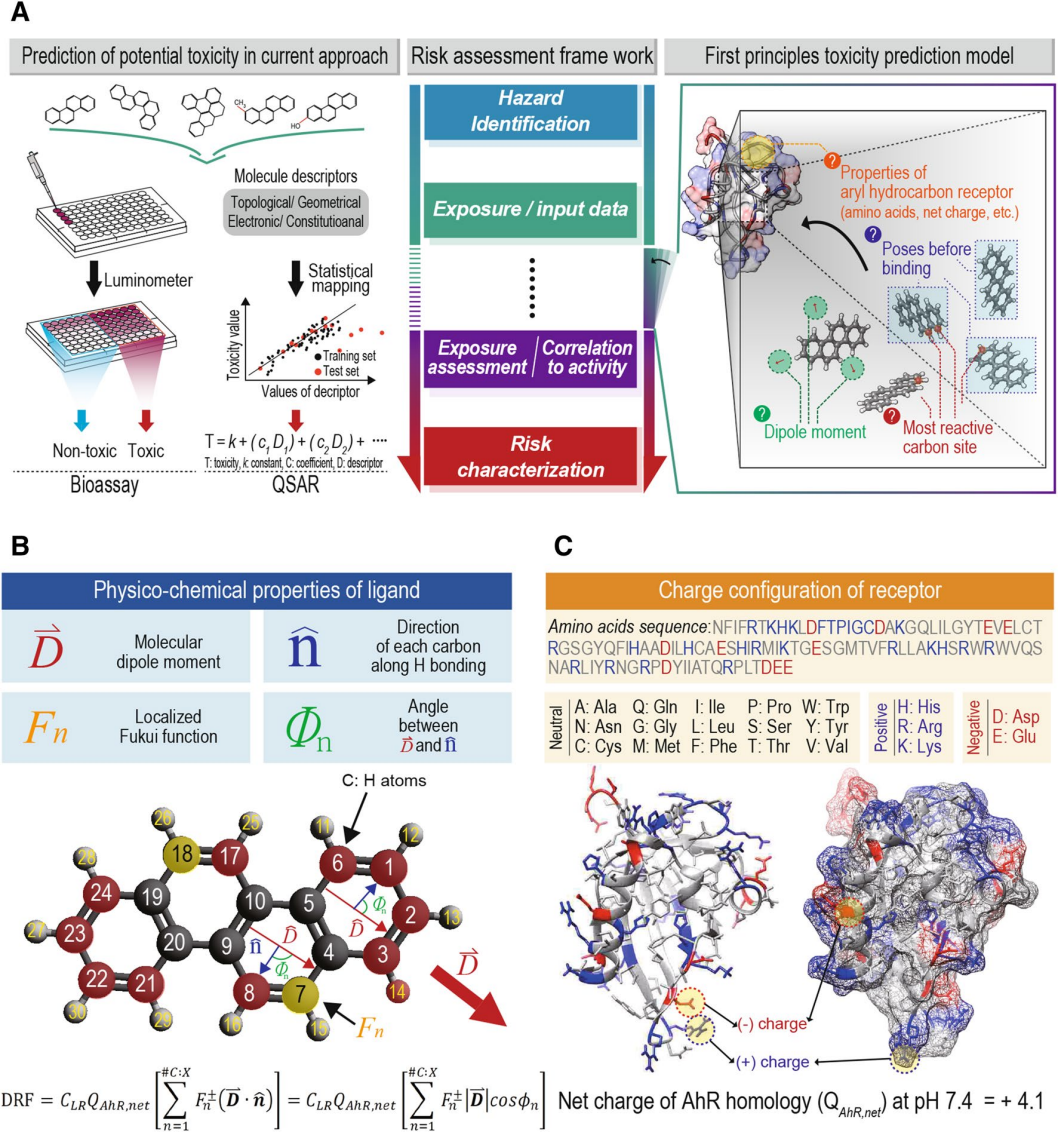
Directional reactive modeling for AhR mediated potency. (**A**) Comparison between contemporary experimental approaches, quantitative structure–activity relationship (QSAR) and first principles potential toxicity prediction model; (**B**) Physico-chemical properties of the model compound, chrysene, for the directional reactive factor (DRF); (**C**) Structural model of aryl hydrocarbon receptor (AhR) with ligand binding sites based on PDB ID: 4F3L and its amino acids sequence which is counted to 107. Net charge of AhR homology (*Q*_*AhR,net*_) is determined to + 4.1.

In ecotoxicology, assessment of toxic potencies has relied primarily on empirical observations of biological responses through in vivo and later developed in vitro systems^7^. Aside from ethical issues involved in testing of whole animals and difficulty of studying threatened or endangered species or non-standard laboratory animals, the rapid upsurge in new chemicals either found in nature or synthesized in industry outpaces capacities of industries and governments to assess risks by use of traditional methods, which makes them impractical for routine screening and testing^8^. In an effort to complement traditional assays and efficiently screen new chemicals, this proliferation of new chemicals has led to increasing attention being given to in silico alternatives to empirical testing, especially in vivo^9^. Quantitative structure–activity relationships (QSAR) is one accepted alternative, in silico predictive method based on linear free energy models (LFEM) and statistical correlations between structure-related physico-chemical properties of ligands in form of molecular descriptors (numerical quantities) and previously experimented bioactivity data. Multiple linear regression equations developed by the QSAR approach are then used to extrapolate target endpoints, such as toxic potencies of new chemicals (Fig. 1A)^10–12^. LFEMs include predictors of hydrophobic, electrical, and steric parameters. When structure-related properties are however, not linearly correlated to bioactivities, statistical inferences of target endpoints during semi-empirical modeling are not applicable^13–15^. Alternatively, recently, molecular docking models, based primarily on steric considerations, are being used to analytically estimate the binding affinity of ligands with receptors^16,17^.

In a previous study, we introduced the directional reactivity factor to predict bioavailabilities of polychlorinated biphenyl congeners (PCBs) based on first principles. But physico-chemical information of participating receptors, such as electronic charge state was not considered in the predictive equation, which makes it limited for predicting receptor-dependent bioactivity^18^. Here, we report a novel, advanced directional reactive (DR) model that illustrates how physical properties of a ligand interplay to constitute optimal environments for biochemical interactions with respective receptors (Fig. 1A). The DR approach identifies three, stepwise key parameters and optimal conditions affecting ligand-receptor activity: (1) dipole-driven electrostatic interactions between ligands and receptors; (2) molecular orientation induced by physical ligand-receptor interaction and (3) resulting directional configuration of reactive sites in ligands. As a proof of concept, the DR model was applied to a ligand class containing homologues of polycyclic aromatic hydrocarbon (PAH), chrysene interacting with cytosolic protein, the aryl hydrocarbon receptor (AhR). Variation of the AhR-mediated toxic potencies across chrysene and its derivatives was well reproduced by the predictive model based on first-principles.

## Results

### Directional reactive modeling

For modeling interactions between ligands and receptor proteins, we explored two hypotheses; (a) different binding activities of various ligands with the same kind of receptor (AhR in this study) would solely depend on the ligand’s physico-chemical properties and (b) different binding activities would be resulted from the interaction of ligands with common properties of the receptor. These hypotheses suggest that a proper identification of physical properties of the ligand compounds and their interplay configuring the optimal interaction conditions with the common receptor would provide a probabilistic estimation of the ligand-specific binding activity. Then, the estimated ligand-receptor binding activity can be verified by comparison with statistical observation of receptor-mediated toxic potency.

When a ligand (drug or xenobiotic) is introduced into the cytosol of cells, it encounters various forces such as electrostatic, hydrogen bonding, π-π stacking, and van der Waals forces^19^. Among these, electrostatic interaction is often the dominant force at large ligand-receptor separation before binding, while others are relatively weak or working over only small distances^20^. This electrostatic interaction is mediated by dipole moments of ligands and charge state contributed from charged amino acids in a receptor. Considering larger molecular weights of receptor proteins than that of ligand molecule, charges on receptors can be assumed to be fixed in space and result in rotation of the ligand to make its dipole moment vector align along the line of force action, while the ligand moves toward the receptor. This process would determine the relative orientation of a ligand to “active binding” sites of the receptor. Another necessary condition to be considered for optimal reactions, resulting in ligand-receptor binding, is distribution of “frontier molecular orbitals (FMOs)” of a ligand, that is, the highest occupied molecular orbital (HOMO) and the lowest unoccupied molecular orbital (LUMO). Spatial distributions of HOMO and LUMO are related to charge densities in FMOs and thus, suggest which constituent atoms in a molecule serve as preferred sites for nucleophilic or electrophilic interactions with charged amino acids in a receptor. The DR model employs the atom-condensed Fukui function (*F*) as a FMO reactivity indicator. This approximation is valid for most cases of negligible orbital relaxation^21^. The optimal ligand-receptor reaction would then require the more reactive sites to be located at the front of the ligand, which is governed by the “dipole moment-driven alignment” (Fig. 1B). Considering these physico-chemical processes, we developed directional reactivity factors (DRFs), which describe favorable orientation configurations between ligands and receptors, given the charge-dipole interaction (Eq. 1).

1 $$ {\text{DRF}} = C_{LR} Q_{R, net} \left[ {\mathop \sum \limits_{n = 1}^{\# AX} F_{n}^{ \pm } \left( {\user2{\overset{\lower0.5em\hbox{$\smash{\scriptscriptstyle\rightharpoonup}$}} {D} } \cdot \hat{\user2{n}}} \right)} \right] = C_{LR} Q_{R,net} \left[ {\mathop \sum \limits_{n = 1}^{\# A:X} F_{n}^{ \pm } \left| {\user2{\overset{\lower0.5em\hbox{$\smash{\scriptscriptstyle\rightharpoonup}$}} {D} }} \right|\cos \phi_{n} } \right] $$

where *Q*_*R,net*_ is the receptor’s net charge contributed from charged amino acids, *F*^+^ (*F*^*-*^) is the nucleophilic (electrophilic) Fukui function of each terminal atom bonded with hydrogen or other functional groups (#*A:X*) in the ligand, $$\user2{\overset{\lower0.5em\hbox{$\smash{\scriptscriptstyle\rightharpoonup}$}} {D} }$$ is the ligand’s dipole moment vector, and $$\hat{\user2{n}}$$ is the bonding direction of the terminal atom. The correlation coefficient *C*_*LR*_ is $$1/4\pi \varepsilon_{o} \varepsilon_{R} r$$ that represents Coulomb constant, relative permittivity of receptor cytosol ($$\varepsilon_{R}$$), and the inverse proportionality of charge-dipole interaction to the intermolecular distance (*r*). This coefficient becomes constant under the condition of equal ligand-receptor distance for the same type of receptor.

The DRF equation takes into account contributions of all atom-condensed Fukui functions along the line of action ($\hat{\user2{D}}$). This describes reactive sites located behind the molecule (π/2 < *ϕ*_*n*_ < 3π/2) with respect to the line of action that would reduce reaction affinity with the receptor. The use of either *F*^+^ or *F*^*-*^ in the DRF calculation depends on the charged state of the responsive receptor. These quantities are computationally obtainable using the homology modeling method and the density functional theory (DFT)^22,23^.

### DRF as an indicator of ligand-binding reactivity

The DR model was applied to predict AhR mediated toxic potencies of homologues of chrysene by examining the DRF, which represents the degree of optimal reaction configuration toward ligand-receptor binding. Cytosolic AhR is a widely used ligand-activated transcript factor regulating expression of CYP450, xenobiotic metabolizing enzymes, which occur in diverse species and cell types^24^. Chrysene is listed as one of 16 priority PAHs by the US Environmental Protection Agency (US EPA) and has a variety of homologues^25^. The model compounds included 4 groups of differently modified chrysene homologues; benzo-, dibenzo-, methylated, and hydroxylated chrysenes. In each homologue group, 2−3 congeners were targeted to generally encompass homologous variations (Table S1 for full list of chrysene homologues). The net charge of AhR (*Q*_*AhR,net*_) was obtained by building three-dimensional (3D) structures of the ligand binding domain (LBD) from the sequence of amino acids in the rat AhR (GI: 7304873 in the NCBI sequence database) using SWISS-MODEL^26^. The 3D AhR homology structure is composed of 107 amino acids in total and contains 5 histidines (His or H), 10 arginines (Arg or R), and 5 lysines (Lys or K), which possess positive charges at pH 7.4 (acidity condition of the in vitro bioassay in this study), and 5 aspartates (Asp or D) and 6 glutamates (Glu or E) of negatively charged acids (Fig. 1C and Figure S1). These charged amino acids result in a total net charge of + 4.1 at pH 7.4 (Figure S2 and Table S2 for details of net charge calculation)^27^.

**Figure 2:**
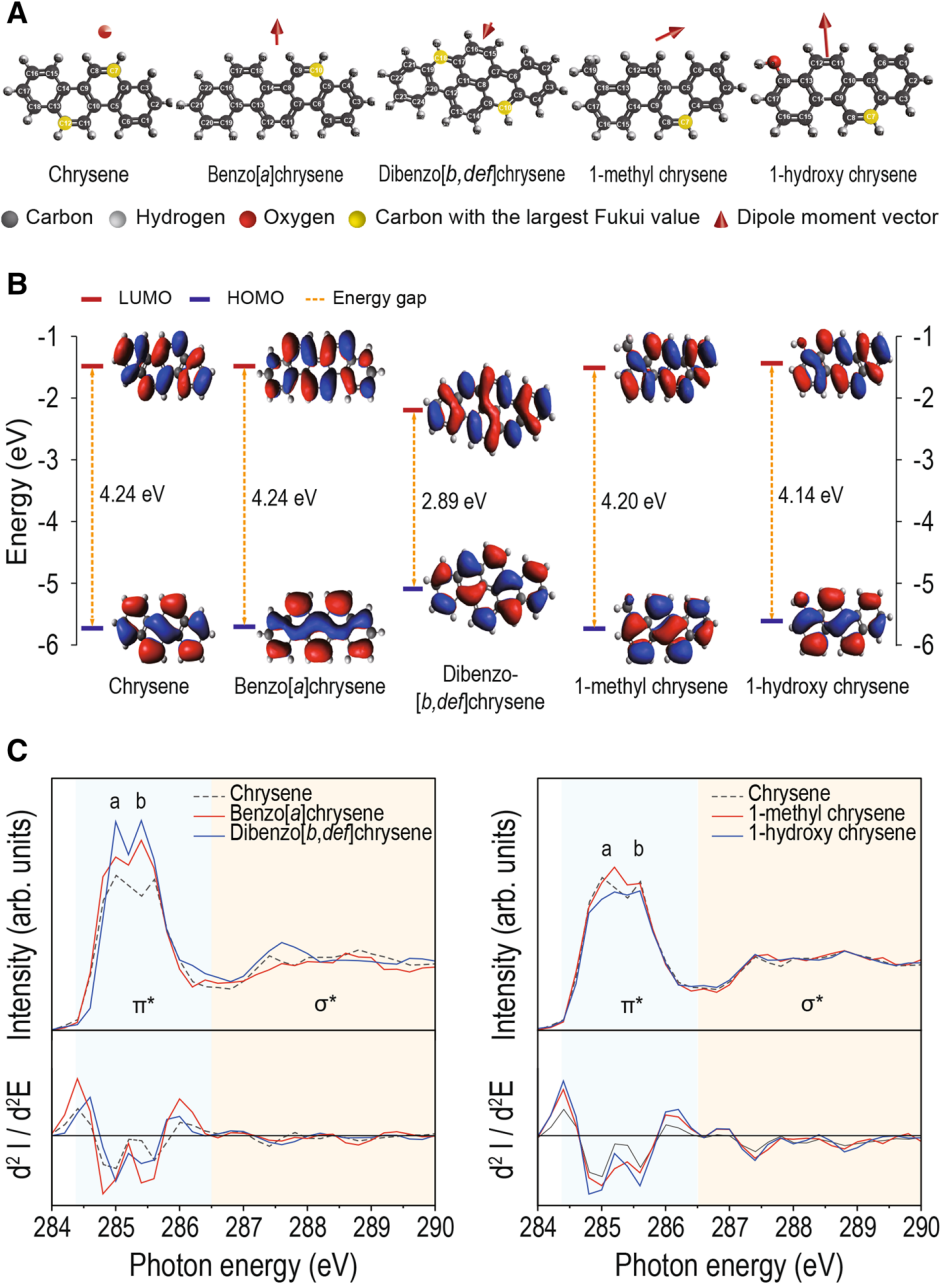
Molecular orbital structures of chrysene and its homologues (benzo, dibenzo, methyl and hydroxy chrysenes). (**A**) Change in dipole moment by addition of a benzene ring and functional group (methyl- and hydroxy-) of chrysene. Carbon atoms with the largest Fukui value in each chrysene homologue are highlighted in yellow and the red arrow denotes the direction and magnitude of dipole moment; (**B**) The HOMO and LUMO energies and corresponding orbital iso-surfaces for the chrysene homologues; (**C**) C 1*s* near-edge X-ray absorption fine structure (NEXAFS) spectra of chrysene homologues collected from pristine samples. Photon energy ranges exhibiting spectral features of carbon atoms bonded with hydrogen or other functional groups, C-H or C-X (peak **a**) and those bonded with neighboring carbon atoms, C–C (peak **b**).

Various physico-chemical properties of chrysenes, such as HOMO, LUMO, and molecular dipole moment, were determined by ab initio DFT calculations with Becke three-parameter Lee–Yang–Parr (B3LYP) exchange functional and the polarized triple-zeta valence (def2-TZVPP) basis set. The atom-condensed Fukui function was determined from the DFT-calculated Hirshfield charge population^28^. Due to structural symmetry, when the chrysene base molecule is modified by electrophilic methyl and nucleophilic hydroxyl groups while the base molecule has a weak dipole moment almost perpendicular to the molecule plane, the dipole moment changes dramatically, both in direction and magnitude (Fig. 2A, Table 1, and Figure S3). The additive rings (benzo- and dibenzo-chrysenes) also result in rotation of dipole moment with small increase in magnitude. The hydroxy chrysene group has the largest dipole moment followed by the methyl group. The dipole moments of benzo and dibenzo groups are comparable to that of the base molecule. The electrophilic methyl and nucleophilic hydroxyl groups substantially increase the dipole moment magnitude. Alternatively, frontier orbitals (HOMO and LUMO) responsible for intermolecular interaction are additionally contributed from orbitals around carbon sites in benzo- and dibenzo-chrysenes (Fig. 2B and Figure S4).

**Table 1:** Structural and electronic configuration parameters of the chrysene homologues and the potential toxicity predicted by directional reactivity factor (DRF).

Compounds	HOMO (eV)	LUMO (eV)	HOMO–LUMO energy gap (eV)	Dipole moment magnitude (10^2^ Debye)	Fukui value	Carbon atoms with the largest Fukui value	Location of carbon* to AhR	Directional reactivity factor (DRF)
					***F***^**+**^			
**Chrysene**	− 5.75	− 1.51	4.24	0.03	0.068	7C, 12C	P	− 0.02
Benzo[*a*]chrysene	− 5.73	− 1.50	4.24	3.30	0.062	10C	B	− 2.98
Benzo[*b*]chrysene	− 5.43	− 1.94	3.49	1.89	0.056	8C	F	1.60
Benzo[*c*]chrysene	− 5.77	− 1.59	4.18	10.10	0.060	10C	F	9.75
Dibenzo[*b*,*def*]chrysene	− 5.11	− 2.22	2.89	0.03	0.062	10C, 18C	B	− 0.02
Dibenzo[*def*,*p*]chrysene	− 5.36	− 1.97	3.39	3.43	0.059	10C	B	− 3.57
1-methylchrysene	− 5.69	− 1.49	4.20	39.58	0.069	7C	F	6.79
2-methylchrysene	− 5.68	− 1.47	4.21	61.40	0.067	7C	F	27.97
3-methylchrysene	− 5.67	− 1.44	4.23	57.52	0.067	7C	F	13.66
1-hydroxychrysene	− 5.56	− 1.43	4.14	131.50	0.070	7C	B	− 33.19
2-hydroxychrysene	− 5.66	− 1.49	4.17	145.70	0.069	7C	B	− 51.44
3-hydroxychrysene	− 5.62	− 1.48	4.14	94.04	0.059	12C	B	− 58.75

*The carbon with the largest Fukui value; forward (F), perpendicular (P), and backward (B) to the AhR.

Carbon in the methyl group does not possess LUMO and addition of the hydroxyl group suppress LUMO from nearby carbon atoms. The influence of molecular modification on frontier orbitals is supported by measurements of C 1*s* near-edge X-ray absorption fine structure (NEXAFS) (Fig. 2C). Two spectral features about 284.8 and 285.6 eV correspond to C 1*s* → LUMO transitions from carbon atoms bonded with hydrogen or other functional groups (peak **a**) and those bonded with neighboring carbon atoms (peak **b**)^29^. Addition of a methyl group increases the spectral weight of a peak at the higher energy than LUMO of chrysene base molecule (284.8 eV) and thus the π* state of carbon in the methyl group does not appear in the LUMO iso-surface. Suppression of the LUMO state due to the presence of the hydroxyl group is also supported by a decrease in the spectral weight of peak **a,** although there is no loss of a carbon atom through hydroxylation (Figure S5 for C 1*s* NEXAFS results for all chrysene homologues).

Using DFT-calculated properties and total net charge of the AhR, DRF values for all 12 homologues of chrysene were obtained (Table 1). The charge-dipole interaction would occur in the cell cytosol containing dissolved ions such as Na^+^, K^+^, and Cl^-^, not in free space^30^. Results of a recent study suggested that these dissolved ions are attracted by and trapped in the vicinity of the charged amino acids in cytosolic proteins^31^. That is, Na^+^ and K^+^ ions are bound to negative Asp and Glu residues while Cl^-^ ions are to positive Arg, Lys, and His residues. After being introduced into the cell cytosol, the ligand thus recognizes the electrostatic potential by these ions surrounding the charged amino acids, which can be approximated to opposite charge of the AhR protein (-*Q*_*AhR,net*_). The dipole moment vector and Fukui functions resulting in the DRFs are selected in this regard. The most striking result is that hydroxy-chrysene has the smallest DRF values despite their strongest dipole moments. This is because carbons with the large Fukui functions are located on the opposite side of the ligand from the receptor (Table 1 presents as an example, the location of carbon with the largest Fukui value). Methyl chrysene exhibited the largest DRF values, followed by benzo-chrysenes. In each homolog group, DRF values depend on the dipole moment and relative location of reactive carbons to the dipole orientation. The DRF shows the large value (27.97) for 2-methylchrysene (2MC) and decreases to 6.79 for 1-methylchrysene (1MC). In the case of benzo[*c*]chrysene (BcC), the DRF is larger than that of 1MC, because of the favorable distribution of reactive carbon sites despite the smaller *F*^+^ and dipole moment.

### AhR-mediated toxic potency of chrysene homologues

**Figure 3:**
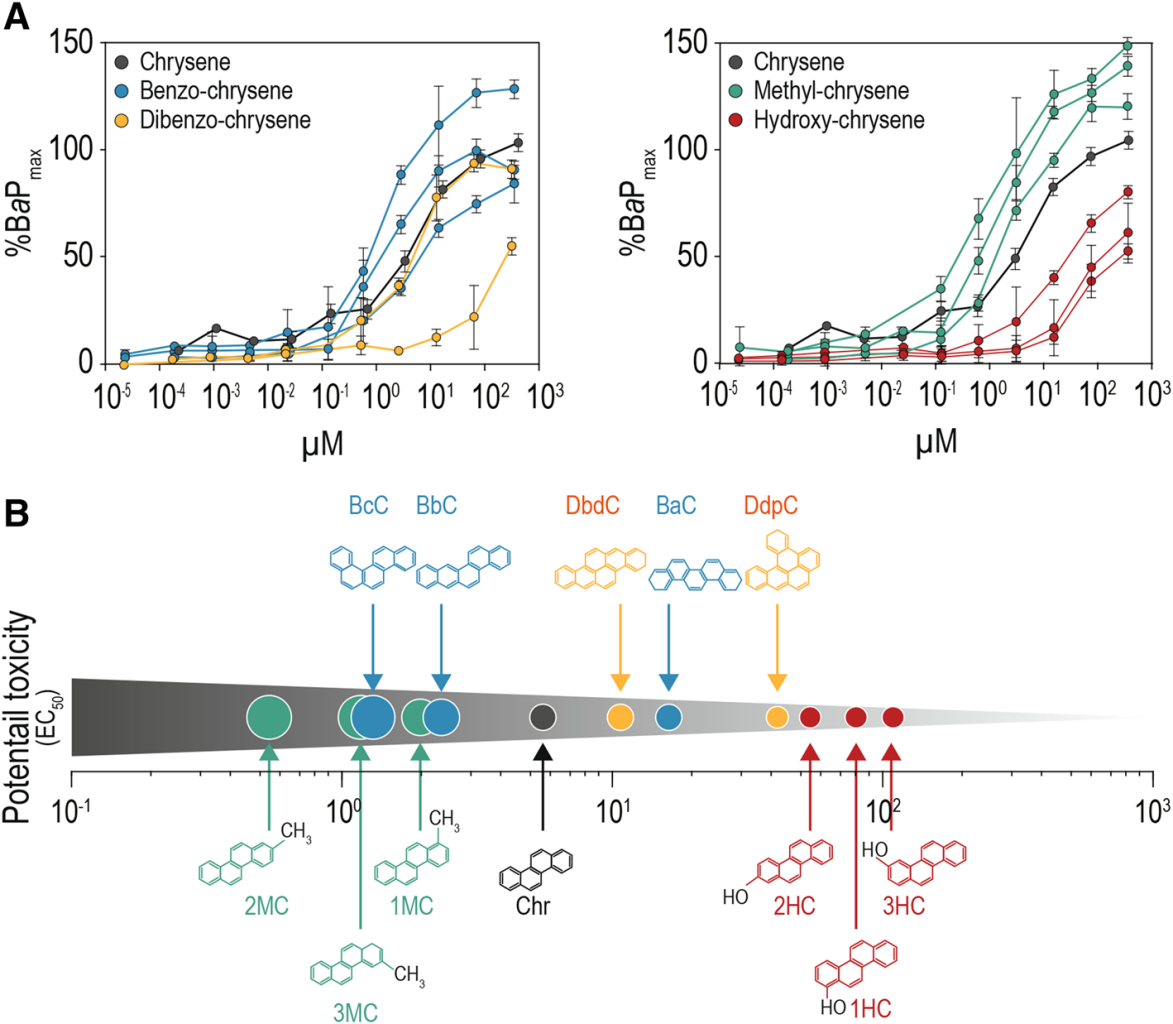
Experimental AhR-mediated potencies of chrysene homologues. (**A**) Dose-dependent activation of chrysene base compound and its homologues; benzo (*n* = 3), dibenzo (*n* = 2), methyl (*n* = 3), and hydroxy chrysenes (*n* = 3). Relative responses were normalized against the positive control (benzo[*a*]pyrene at the following concentrations; 100, 20, 4, 0.8, 0.16, 0.032, 0.0064, 0.00128, 0.000256, or 0.0000512 μg mL^−1^; set as 1.0 of potential toxicity). Dose expressed as nmol chrysene homologues/mL media (μM) present in the test well. EC_50_ are means of three or four independent experiments. Error bars represent the standard error; (**B**) The order of potential toxicity of chrysene homologue based on EC_50_ calculated for H4IIE-*luc* transactivation assay. Examined chrysene homologues were ranked in descending order of potential toxicity. The methyl chrysene group exhibits the highest potency followed by benzo chrysene one while dibenzo chrysene and hydroxy-chrysene groups show similar or lower potencies than other chrysene homologue groups.

If the DR model describes the optimal configuration for ligand-receptor reaction properly, the DRF would be one promising indicator accurately estimating ligand-specific bioactivity. For robust validation of the DR model, a homogeneous set of AhR-mediated toxic potencies for chrysenes were examined experimentally by use of the in vitro, H4IIE-*luc* transactivation assay and compared with DRF values calculated from first-principles. The experimental dose–response curves for the groups of similarly structured chrysenes are presented (Fig. 3A). AhR-mediated potencies of all chrysene homologues are evidenced in the range of 51–147%BaP_max_, which indicated significant potential to cause toxicity and suggests selected target chemicals are as potent as benzo[*a*]pyrene, which is a well-known in vitro agonist of the AhR. By group, methyl chrysene exhibits the greatest potency, followed by benzo-chrysene, while dibenzo-chrysene and hydroxy chrysene both exhibited similar or lesser potencies, compared to that of the base compound of unsubstituted chrysene.

For a detailed comparison with the DRF value for individual compounds, the half-maximal effective concentration (EC_50_) values were calculated (Fig. 3B and Table 2). Among chrysene homologues tested, 2MC turns out to have the greatest toxic potency (EC_50_ = 6.6 × 10^−1^ μM) and 3-hydroxychrysene (3HC) was the least potent (EC_50_ = 1400 × 10^−1^ μM). Different EC_50_ values were also observed within each homologue group. The EC_50_ values of benzo[*a*]chrysene (BaC), benzo[*b*]chrysene (BbC), and BcC are 200 × 10^−1^ μM, 28 × 10^−1^ μM and 13 × 10^−1^ μM, respectively, despite having the same molecular formula, mass, and octanol–water partition coefficient (Log K_ow_; hydrophobicity). For the dibenzo-chrysene group, differences in EC_50_ values were between dibenzo[*b,def*]chrysene (DbdC) (140 × 10^−1^ μM) and dibenzo[*def,p*]chrysene (DdpC) (760 × 10^−1^ μM). A similar trend was observed for the methyl- and hydroxy-chrysene groups. The experimentally derived bioactivity data was consistent with the optimal ligand-receptor reaction predicted by the DR model for toxicity rank among homologue groups and also among congeners within a homologue. This consistency suggests that accurate predictions of ligand-receptor binding and resulting ligand-specific bioactivity requires contributions involving not only physico-chemical properties but also interplay between and among them.

**Table 2:** Comparison of the directional reactivity (DR) model with other predictive models and experimental potential toxicity data.

Chemical	In vitro bioassay	QSAR^a^	Docking model^b^				Directional reactivity factor (DRF)
	H4IIE-*luc* EC_50_	*Daphnia magna* EC_50_	Free energy (kcal mol^−1^)	Binding distance to LBD (Ǻ)			
	(μM)	(μM)		Total	H285^c^	F318^d^	
2-methylchrysene	6.6 × 10^−1^	7.4 × 10^−1^	− 7.1	6.3	3.0	3.3	28.0
3-methylchrysene	11 × 10^−1^	7.4 × 10^−1^	− 10.5	6.3	3.0	3.4	13.7
Benzo[*c*]chrysene	13 × 10^−1^	5.4 × 10^−1^	− 8.0	7.4	3.6	3.8	9.8
1-methylchrysene	19 × 10^−1^	7.4 × 10^−1^	− 9.8	6.6	3.1	3.4	6.8
Benzo[*b*]chrysene	28 × 10^−1^	5.4 × 10^−1^	− 6.6	22.8	9.1	13.8	1.6
**Chrysene**	72 × 10^−1^	9.1 × 10^−1^	− 9.7	6.6	3.2	3.4	− 0.02
Dibenzo[*b*,*def*]chrysene	140 × 10^−1^	4.2 × 10^−1^	− 6.1	23.2	9.2	14.0	− 0.02
Benzo[*a*]chrysene	200 × 10^−1^	5.4 × 10^−1^	− 5.6	25.1	10.6	14.5	− 3.0
Dibenzo[*def*,*p*]chrysene	760 × 10^−1^	4.2 × 10^−1^	− 7.8	33.0	18.4	14.6	− 3.6
2-hydroxychrysene	590 × 10^−1^	32 × 10^−1^	− 8.0	31.9	17.7	14.2	− 51.4
1-hydroxychrysene	870 × 10^−1^	32 × 10^−1^	− 8.9	32.3	17.7	14.6	− 33.2
3-hydroxychrysene	1400 × 10^−1^	32 × 10^−1^	− 8.1	6.7	2.9	3.8	− 58.8

^a^Predicted data from VEGA-QSAR.

^b^Galaxydock.

^c^Histidine 285.

^d^Phenylalanine 318 in the AhR homology.

### Comparison of predicted potencies: DR model versus current in silico models

DRF values and experimental toxic potency data (inverse of EC_50_) were compared with the toxicity-relevant characteristics predicted by currently used in silico predictive models; QSAR (inverse of the median effective concentration, 1/EC_50_), a structure-based statistical regression method, and molecular docking models (free energy and binding distance), a structure-based analytical approach searching for the best ligand-receptor binding pose in terms of binding free energy^32,33^. Target toxicants can be input as the unambiguous notation of the structure such as SMILES in VEGA-QSAR. The results of predictive EC_50_ were obtained from the Instituto di Ricerche Farmacologiche Mario Negri (IRFMN) toxicity model (*Daphnia magna* acute) based on the training sets including aromatic hydrocarbons^34^. The same AhR homology used in the DR model was employed for calculations with docking models. Overall, the results suggest that both QSAR and molecular docking models do not accurately estimate the experimental potencies determined by use of in vitro bioassays, say anti-empirical data.

**Figure 4:**
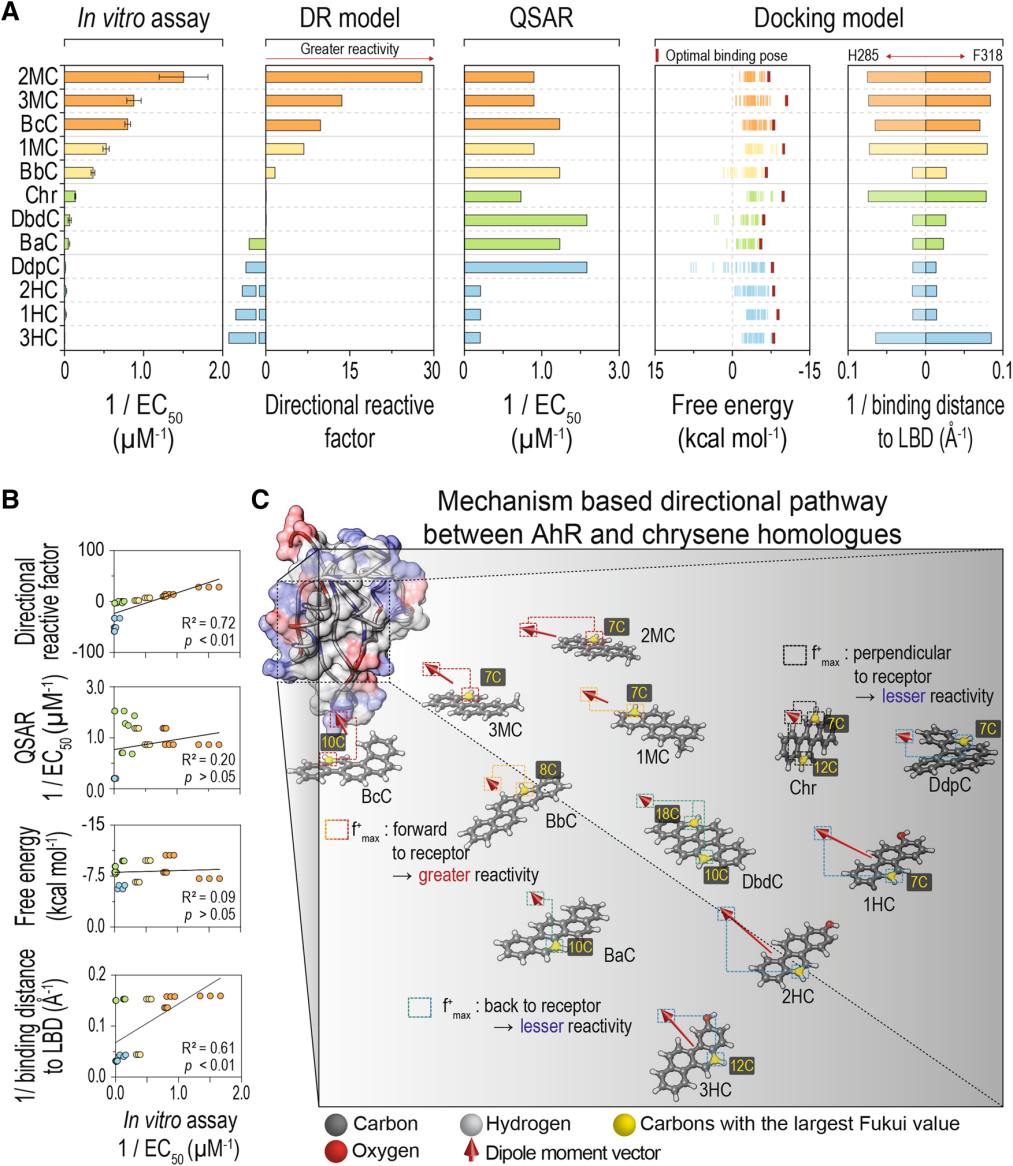
Prediction efficiency of DR model and other in silico assays. (**A**) Experimental potency results of in vitro bioassay (H4IIE-*luc*) and predicted toxicity-relevant characteristics by in silico models (QSAR, molecular docking model, and the DR model introduced here). The QSAR and the docking model calculations were made using VEGA-QSAR and GalaxyDock, respectively. Each toxicant in the graph was sorted in descending order of 1/EC_50_ value. (**B**) Predicted versus observed values represented from each method and the regression coefficient, R^2^ with significance (*p*). (**C**) Visualization of first principles prediction model for AhR-mediated potency of chrysene homologues before their binding to AhR.

The QSAR model predicted greatest toxic potency of dibenzo- and benzo-chrysene groups followed by chrysene base compound and methylated chrysenes. Different experimental toxicities within each homologue group are also not reproduced, rather the similar degree of toxicity among congeners was observed (benzo-chrysene: 5.4 × 10^−1^ μM; dibenzo-chrysene: 4.2 × 10^−1^ μM; methyl-chrysene: 7.4 × 10^−1^ μM; hydroxy-chrysene: 32 × 10^−1^ μM) (Fig. 4A and Table 2). This results in statistically insignificant correlations between experimental 1/EC_50_ and QSAR-estimated 1/EC_50_ (R^2^ = 0.20, *p* > 0.05), which implies considerable uncertainty in prediction accuracy of toxicity (Fig. 4B). Unlike the QSAR approach, the docking analysis predicts different toxic potencies of congeners in each homologue group and high binding affinity behaviors (i.e., low free energy and short binding distance) of the group of methylated chrysene, which was consistent with empirical toxicity data (Fig. 4B and Figure S6).

Chrysene homologues composed of benzene rings (benzo- and dibenzo-chrysene groups) exhibited lesser free energies than those of methyl- and hydroxy-chrysene groups. Another notable feature is that hydroxy-chrysenes show comparable free energy to methyl chrysenes while experimental toxic potency due to addition of the hydroxyl group is least among chrysene homologues tested. Considering the fact that hydroxylated benzene compounds are usually known as metabolites by degradation or metabolism in abiotic and biotic responses, the docking model seems to overestimate toxicities of these model compounds (Table S3 presents a result of potential toxicity from other in silico models to predict toxic potencies)^35^. The statistical correlation between calculated optimal binding affinity and experimental 1/EC_50_ values is found to be insignificant (R^2^ = 0.09, *p* > 0.05) (Fig. 4B). Meanwhile, although a positive correlation between 1/EC_50_ and total binding distance between the ligand and either of two binding domains (H285 and F318) was observed (R^2^ = 0.61, *p* < 0.01), the short binding distances calculated for chrysene and 3HC compounds are inconsistent with experimental toxicity results.

Discrepancies of experimental bioactivity data from toxicity-relevant characteristics predicted by current in silico models are not unexpected. In fact, QSAR approaches are based on algorithms composed of a linear regression relationship between logarithms of aquatic toxicity of known chemicals and their structural molecular descriptors (training set). Effective toxic concentrations of individual chemicals were estimated by use of regression equations after training and the measure or calculated values of corresponding chemical from the descriptors^36^. As a consequence, the congeners in each homologue group with the same molecular weight shows the same toxic potency according to the QSAR model.

## Discussion

Toxicity testing has become central to hazard assessments of (in)organic substances associated with causal exposure in environments. Thus, accurate evaluation of adverse effects of chemicals on bio-organisms have been a profound problem in environmental and pharmaceutical toxicology^37^. Development of a model to reliably predict bioactivity complementing traditional bioassays is directly related with a fundamental question about how a chemical trigger the ligand-receptor binding that results in the following expression of xenobiotic metabolizing genes. Current computational approaches have focused on statistical correlations between structure-based physico-chemical properties and the observed bioactivity endpoints using multiple linear regression (i.e., QSAR), and characteristics of the ligand-receptor binding after minimization of free energy of binding (i.e., molecular docking)^38–40^. However, here, we have shown that structural similarity does not always guarantee that bioactivities will also be similar, but small structural differences, like different functionalization sites between 1MC and 2MC, can result in dramatic differences in toxic potency. Furthermore, since long-range dipole-charge interactions predetermine orientation and relative distribution of ligand’s reactive sites before binding to the receptor, the best ligand-receptor binding pose with the minimum free energy must not be always plausible.

The DR model suggests that individual physico-chemical properties of specific ligands do not contribute independently to binding, but interplay between them is also important. This kinetic approach begins with consideration of the “molecular dipole moment” that is sensitive to functionalization, as the main property of ligand interacting with a receptor (mediated by trapped cytosolic ions) after a ligand is introduced into the cell cytosol. However, the favorable binding environment is achieved by combined influence from the molecular orientation and relative location of reactive sites driven by “alignment” of the dipole moment (Fig. 4C). In this study, the DRF devised from the molecular level kinetic process could estimate the macroscopic toxic potency of chrysene homologues and therefore serve as a bioactivity indicator of chemicals. Once the amino acid sequence and constituent, charged acids are identified, the DR model can be applied to investigate species-specific bioactivity.

Unlike other current models, this model does not predict toxicity through statistical methods, but predicts the possibility of toxic effects, assuming the pre-conjugation state in the case of toxicants combined with receptors. Based on this model, it was confirmed that the reactivity (Fukui value) of molecules in each ligand group was different depending on the position despite their structural similarity, and the contribution in dipole moment of the ligand was confirmed according to the position of the reactive molecule. Thus, we found that the shape and orientation configuration of toxicants before the ligand-receptor interaction were generally consistent with EC_50_ values. This model was based on a good understanding on the toxic mechanisms and could apply to compounds with different action mechanisms explaining different reactivity of similarly structured chemicals to AhR.

Computational studies have been increasing substantially in the last few years. These computational techniques with toxicity prediction model highly rely on QSAR analysis. While the DR model is an in silico-based approach like QSAR, but it employs more mechanism-driven consideration of toxic reaction rather than statistical inference and extrapolation. The DRF could bring up a topic of understanding the more scientifically robust basis for risk assessment by providing detailed mechanistic pathways and serve as a complementary to existing QSAR methodology. Using various efficient tools to calculate electronic excitation such as time dependent density functional theory (TDDFT), the DR model have the potential to extend to predict chemical interaction with biomolecules (e.g., fatty acids, DNA, RNA, and micro RNA)^41^. Therefore, further improvement of the DR model will keep pace with mutual advances in molecular biology and computational structural biology and provide a useful tool for discovery of drugs or predictive toxicology.

## Materials and methods

### Selection of model chemicals

As a first step toward identification of the major physical factors affecting toxic potencies, we selected the model chemicals considering four criteria: First, the selection began with a pool of high priority 16 PAHs regulated by the US EPA which have played an exceptionally large role in environmental sciences. Second, candidates were narrowed to 7 PAHs exhibiting acute toxic potency since the DR model does not consider absorption, distribution, metabolism, and excretion, and thus the model compounds should show perceivable variation in the toxic potency for a short period of exposure^42^. Third, for prediction of the binding activity with AhR, the most potent AhR agonist in the environment would be desirable. Finally, various homologues of the selected AhR agonist were added in order to understand how the structural modification of ligand induces different binding activity with AhR and resulting toxic potency. Following these steps, chrysene and its homologues were selected as the model compounds for application of our bio-physical communication model and comparison with experimental results of toxic potency testing. Chrysene homologues cover a broad set of compounds ranging from four- to six-membered benzene to methylated- and hydroxylated benzene; chrysene (Chr), BaC, BbC, BcC, DbdC, DdpC, 1MC, 2MC, 3-methylchrysene (3MC), 1-hydroxychrysene (1HC), 2-hydorxychrysene (2HC), and 3HC. Their full names, providers, and purities are present in the Supplementary information (Table S1).

### Density functional theory calculations

Physical properties of chyrsene and its homologues to be implemented in the DR model were obtained from DFT calculations, which were performed using the ORCA (version 4.1.1) program package^43^. All stages of DFT computations were made by use of B3LYP exchange functional with the def2-TZVPP basis set. Meanwhile, very tight, self-consistent field (SCF) convergence (the energy change is 10^−9^ a.u.) and fine integration grid of 5.0 were employed. The first stage (spin-restricted) geometric optimizations were performed for all structures of the homologues of chrysene. Next, ± *e*, charge was added to each compound as cationic or anionic case for the second stage (spin-unrestricted) optimizations. Based on the corresponding optimized structures, the molecular orbitals, Hirshfeld charge populations, density of orbital states, molecular dipole moments, and vibrational amplitudes for neutral, cationic and anionic states of each chrysene homologue were computed. The atom-condensed Fukui functions for all carbon atoms bonded with hydrogen or other functional groups in each homologue were determined by Hirshfield population analysis^28^.

### C 1s NEXAFS spectroscopy

Since physical properties were determined from the DFT-calculated molecular orbitals of each model compound, the calculated molecular orbital structures were verified through comparison with experimental unoccupied molecular orbital structures measured by NEXAFS spectroscopy. Measurements of C 1*s* NEXAFS spectra were made at the 10D HR XAS KIST beamline of the Pohang Light Source in Korea. All NEXAFS spectra were measured in total electron yield (TEY) mode. After data acquisition, the spectra were energy calibrated with the Π* (C=C) transition at 285.6 eV of highly oriented pyrolytic graphite (HOPG). Samples were tested for radiation damage considering the sensitivity of carbon-based materials to X-ray radiation. Spectra were intensity-normalized to the incoming photon flux, as recorded by an Au mesh.

### H4llE-***luc*** transactivation bioassay for evaluating AhR-mediated potencies and calculation of EC_50_ of homologues of chrysene

H4IIE cell line derived from rat hepatoma has been reported to continuously express cytochrome P450 protein/mRNA. For this study, H4IIE cells transfected with the luciferase (*Luc*) reporter gene plasmid (pGudLuc1.1) were used, originally obtained from Jac Aarts, University of Wageningen, The Netherlands and further developed by John Giesy, University of Saskatchewan^44,45^. Upon ligand binding, AhR in H4IIE-*luc* is activated and translocated to the nucleus, where heat shock proteins are dissociated from the complex and they form a dimer with the AhR nuclear translocator (ARNT) protein. This complex binds to specific DNA sequences with high affinity, the dioxin-responsive element (DRE). The binding to the DRE results in DNA bending transcriptional activation of adjacent responsive genes. The detailed mechanism is provided in Figure S7.

Acute toxic potencies of chrysene homologues were determined based on an AhR-mediated activity using H4IIE-*luc* cells, by the method previously reported^46^. Since PAHs could be degraded by metabolic activity during the H4IIE-*luc* transactivation bioassay with longer exposure time, the 4 h exposure time was chosen for calculation of AhR-mediated toxic potency. After the exposure, the activity results were expressed as relative luminescence units that were quantified using a Victor X3 multi label plate reader (PerkinElmer, Waltham, MA). Benzo[*a*]pyrene was used as a positive control ligand. We converted responses of the H4IIE-*luc* bioassay to the percentages of the maximum response (%BaP_max_) observed for a 50 nM BaP (= 100%BaP_max_). All bioassays were repeated four times in triplicate (Table S4 for detail information of in vitro bioassay conditions). The half effective concentrations for the AhR-mediated effects of individual compounds were determined by use of H4IIE-*luc* bioassays. Compounds with 10 concentrations using fivefold serial dilutions (viz., 100, 20, 4, 0.8, 0.16, 0.032, 0.0064, 0.00128, 0.000256, and 0.0000512 μg mL^−1^) were tested. Estimations of EC_50_ values of target compounds from the dose–response relationships were basically assumed equal efficacy and parallelism between target compounds and reference compound (benzo[*a*]pyrene).

### In silico analysis: quantitative structure–activity relationship (QSAR) and molecular docking model

Prediction capability of current in silico models for chrysene homologues were tested with QSAR model and molecular docking analysis. VEGA-QSAR program and the GalaxyDock program were used for QSAR and molecular docking analysis, respectively. VEGA-QSAR has been performed and showed a large set of toxicological data and endpoints convincing to derive predictive toxicity according to the previous studies^47^. In our case, EC_50_ values were obtained using SMILES notation of VEGA-QSAR and obtained the toxicity data corresponding to *D. magna*.

For molecular docking analysis, the GalaxyDock was used to simulate the optimized configuration of AhR in the presence of model chemicals and determine the binding affinity for receptor-ligand docking at each ligand. The receptor-ligand docking was then simulated using the GalaxyDock in GalaxyWEB^34,48^. For each model chemical, 50 candidate configurations of the ligand-receptor docking were calculated and the configuration with the lowest formation energy was chosen as the optimal binding structure. Two aromatic residues of histidine 285 (H285) and phenylalanine 318 (F318) in the homology modeling were recognized as the most important for ligand binding^49^. The distances between the nearest aromatic ring or residues of chrysene homologues and each of the ligand binding domains were calculated from the predicted binding pose generated by GalaxyDock. For calculations, the three-dimensional (3D) structure of the ligand binding domain (LBD) of the AhR was built from the sequence of amino acids in the rat AhR (GI: 7304873 in the NCBI sequence database) by the validation using the SWISS-MODEL, Rampage web servers, and ProSA-web^26,50^. A crystal structure of 4F3L (sequence identity = 27.8%, residues 252–357) was selected as a template. The structure model, built using Ramachandran plot from Rampage web server, was validated since 98% of the total residues were in the favored region with 2.4% of the residues in the allowed region. Furthermore, results from ProSA analyses showed the z-score for the 3D model to be within the range of score typically found for native proteins of the similar size.

## Supplementary information

Supplementary Information.

## Data Availability

All data are available in the main text or the Supplementary information.
