# Supplementary material for: Influence of ligand’s directional configuration, chrysenes as model compounds, on the binding activity with aryl hydrocarbon receptor
Source: Sci Rep. 2020 Aug 14;10:13821. doi: 10.1038/s41598-020-70704-9 (PMC7428016; doi:10.1038/s41598-020-70704-9)
Supplement: Supplementary file 1 — Supplementary Information. [file 41598_2020_70704_MOESM1_ESM.pdf]

<Scientific Reports>

Supplementary information for

Influence of ligand's directional configuration, chrysenes as model compounds, on the binding activity with aryl hydrocarbon receptor

Taewoo Kim, Juyuan Zhen, Junghyun Lee, Robert Bauer, Changkeun Lee, Bong-Oh Kwon, Keun Hwa Chae, Seongjin Hong, John P. Giesy, Gap Soo Chang*, Jong Seong Khim*

This PDF file includes:

Number of pages: 13

Number of supplementary figures: 7, Figures S1 to S7

Number of supplementary tables: 4, Tables S1 to S4

References

***Corresponding authors:**

E-mail addresses: jskocean@snu.ac.kr, (J.S. Khim); gapsoo.chang@usask.ca (G.S. Chang)

Supplementary Figures

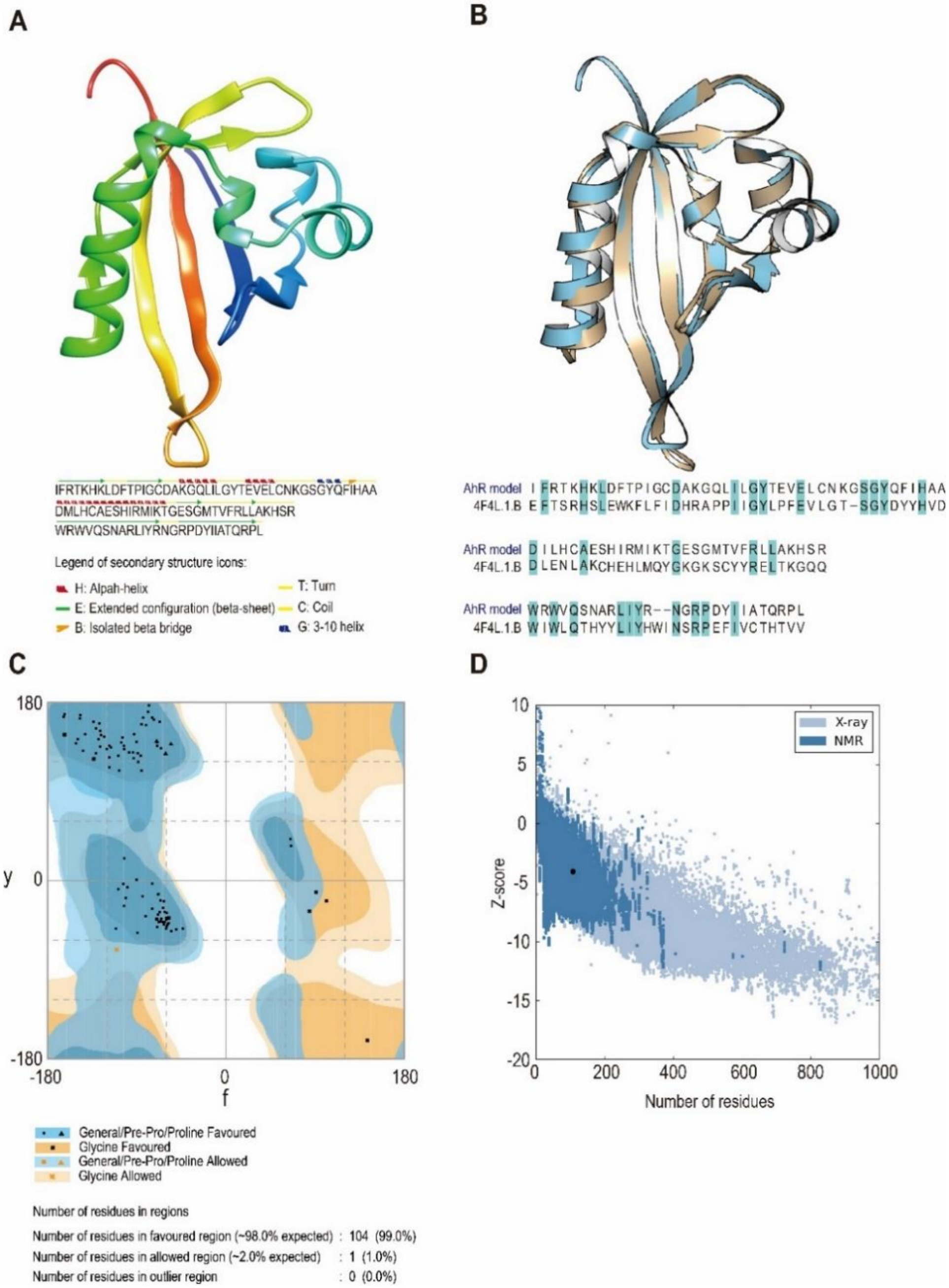

Figure S1. Homology model and quality metrics of AhR LBD. (**A**) Modelled structure of AhR LBD with helices are shown in red, sheets are shown in yellow and loops are shown in green[1]; (**B**) Superimposition of the template (light blue) with the AhR model (light brown) with an RMSD less than 1.0 Å and a structure overlap of 98.13; (**C**) Ramachandran plot showing energetically allowed regions for backbone dihedral angles y against f of amino acid residues in the AhR structure; (D) The ProSA analysis of the generated AhR structure in this study. [2] The figure represents the Prosa-web plot of template 4F3L chain A with a z-score value of − 4.07.

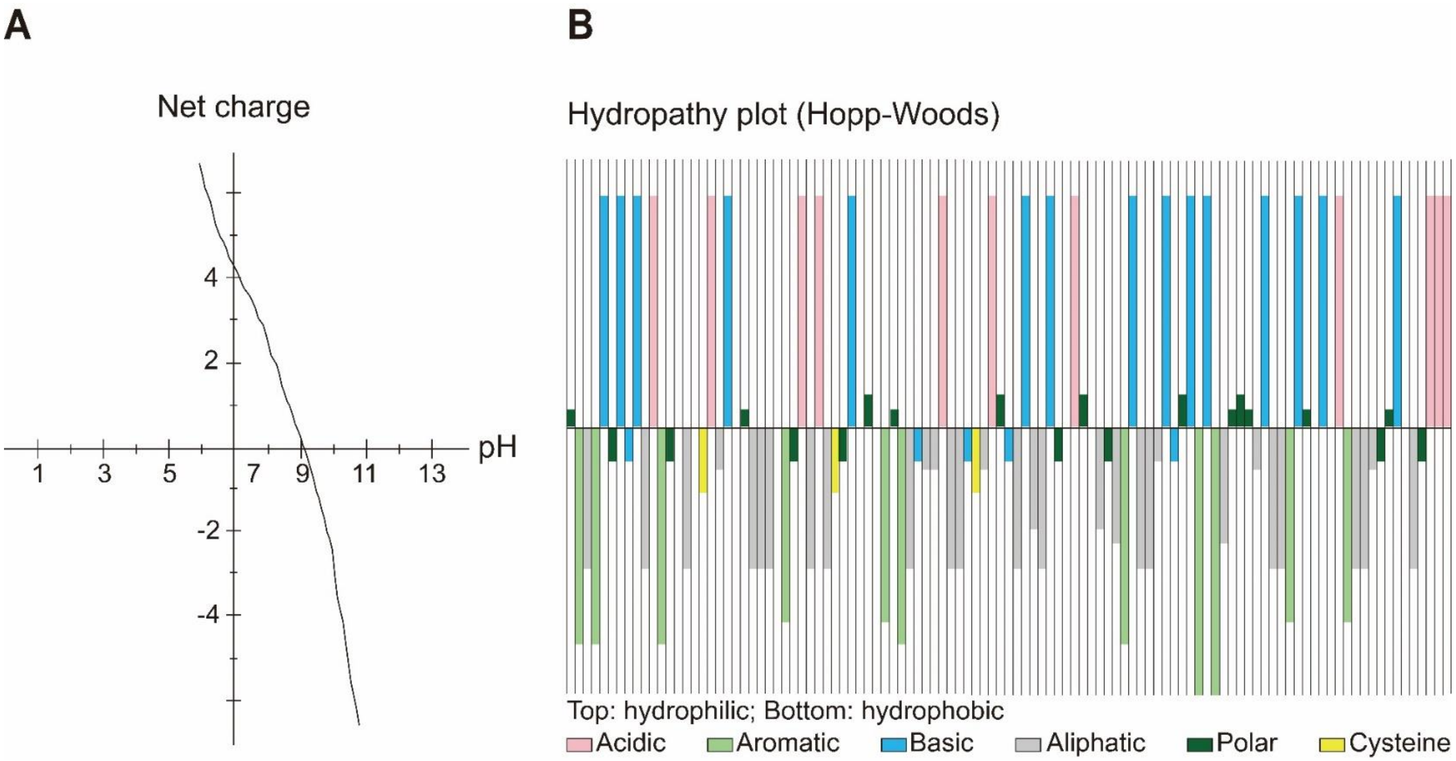

Figure S2. Calculated net charge and hydropathy plot in aryl hydrocarbon homology. (**A**) Expected net charge in AhR homology. AhR homology was expected to have a net charge of 4.1 in pH 7.4[3]; (**B**) Hydropathy plot of AhR homology indicating its physico-chemical properties. The plot showed the degree of hydrophobicity or hydrophilicity of amino acids of AhR. The hydrophobic amino acids accounted for 42.05 of the total sequence in AhR homology, followed by 28.97 % for the hydrophilic amino acids. Basic and acidic were 18.69 % and 10.28 % respectively.

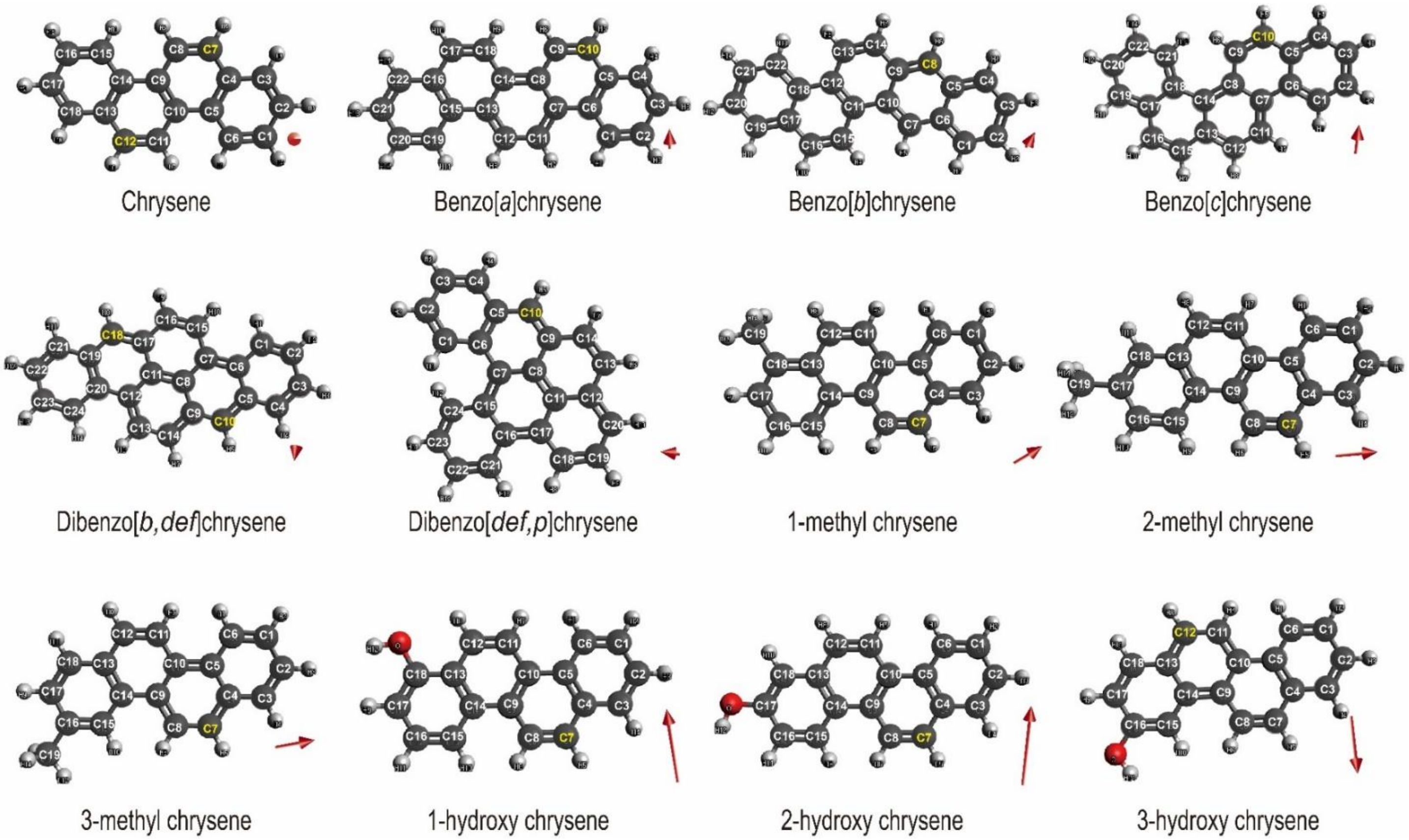

Figure S3. The information of physico-chemical properties in each chrysene homologue. Carbons in structure of chrysene homologues are shown in dark gray, hydrogens are shown in white and oxygen are shown in red color. A carbon with the highest Fukui value of chrysene homologues is shown in yellow, and dipole moments are shown in red arrow. The size of the arrow indicates the quantity of dipole moment.

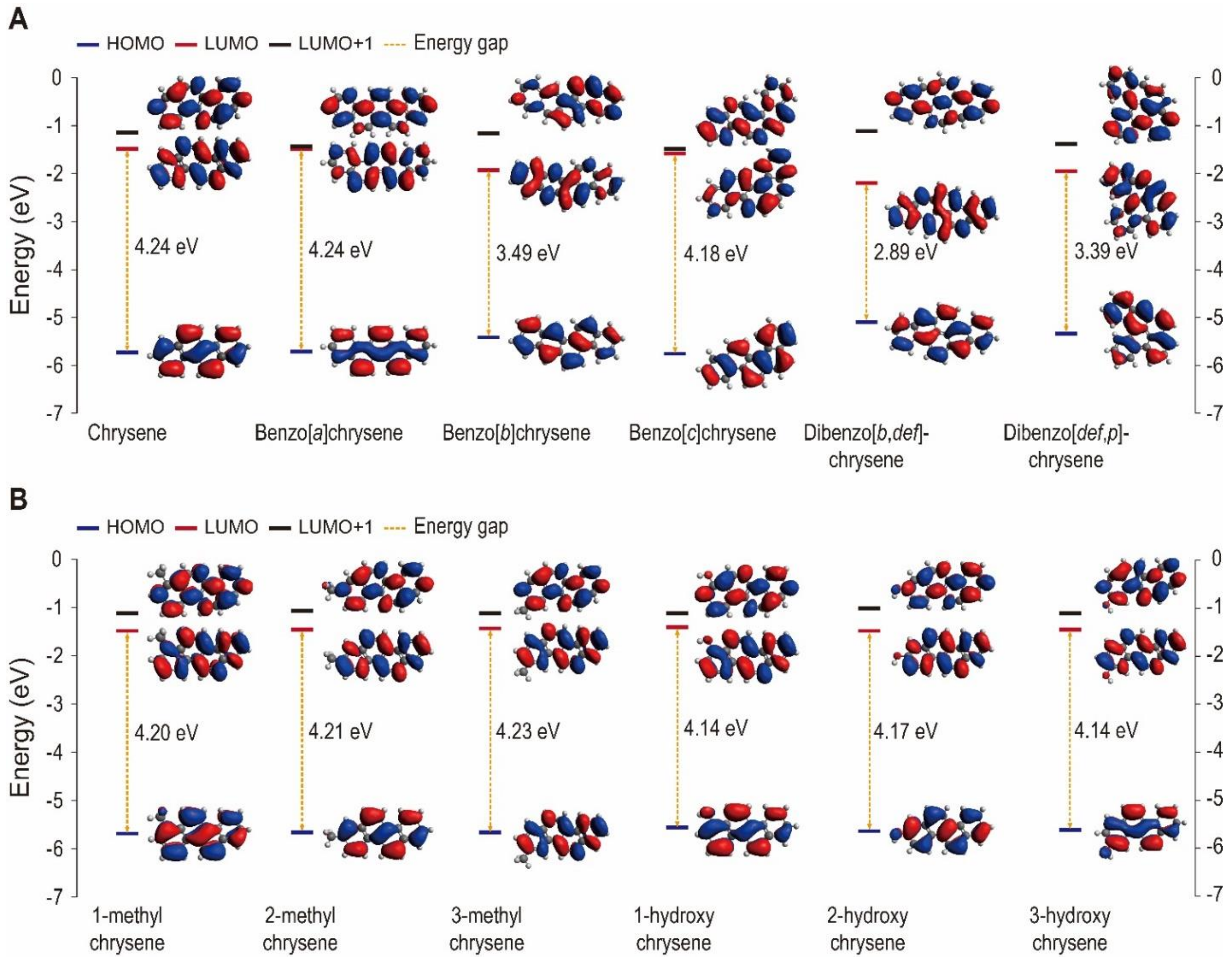

Figure S4. Visualization of the HOMO and LUMO orbital in chrysene homologues. (**A** and **B**) Presentation of the energy levels, HOMO-LUMO gap and orbital composition distribution of the HOMO and LUMO for chrysene and benzo-chrysene. HOMO-LUMO gap energy ranged from 2.89 eV to 4.24 eV. The HOMO-LUMO gap is slightly difference in each congener.

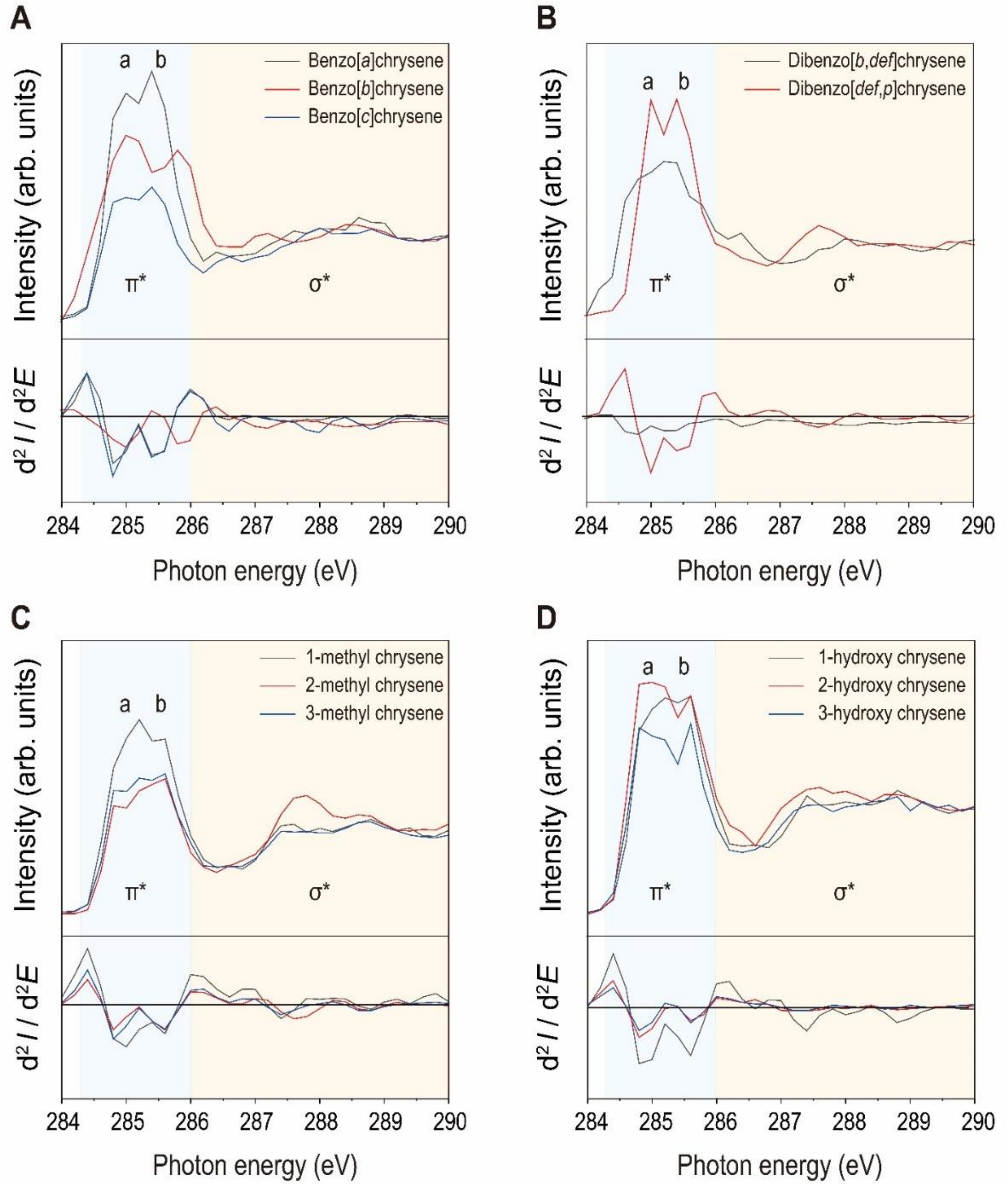

Figure S5. Carbon (1s) NEXAFS spectra of chrysene homologues of chrysene homologues. (**A**) benzo-chrysene, (**B**) dibenzo-chrysene, (**C**) methyl-chrysene, (**D**) hydroxyl-chrysene. The plotted spectra were collected from powder samples. LUMO orbitals were mainly localized around the carbon with hydrogen or other functional groups (peak **a**) and with neighboring carbon (peak **b**)

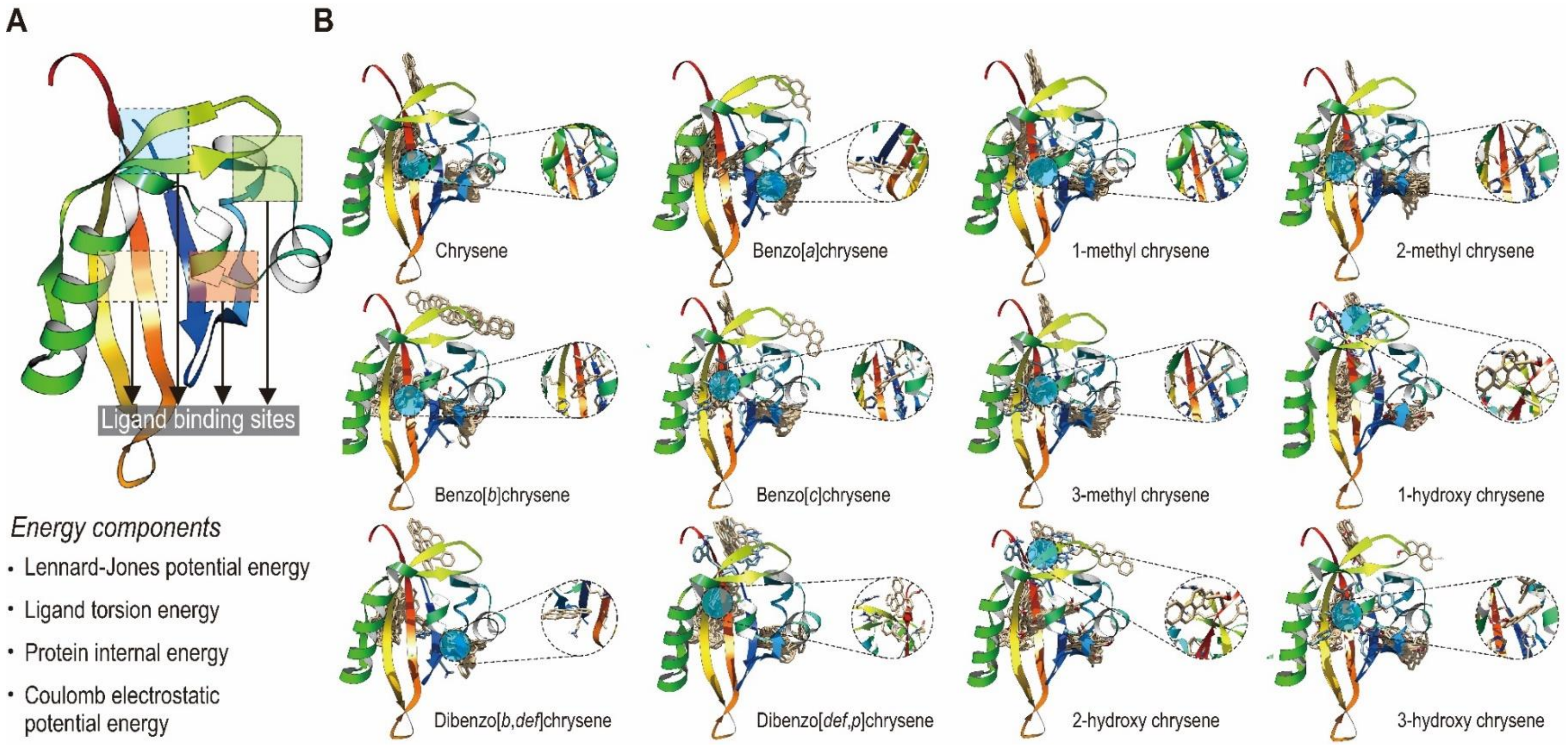

Figure S6. AhR homology and binding poses in the docking model. (**A**) The docking model (Galaxydock) used in this study calculated binding affinity by using various energy components such as Lennard-Jones potential energy, Ligand torsion energy, protein internal energy, and Coulomb electrostatic potential energy[4]; (**B**) The docking results provided fifty possible binding poses between AhR and chrysene homologues and suggests the best optimized binding state which has the lowest binding affinity.

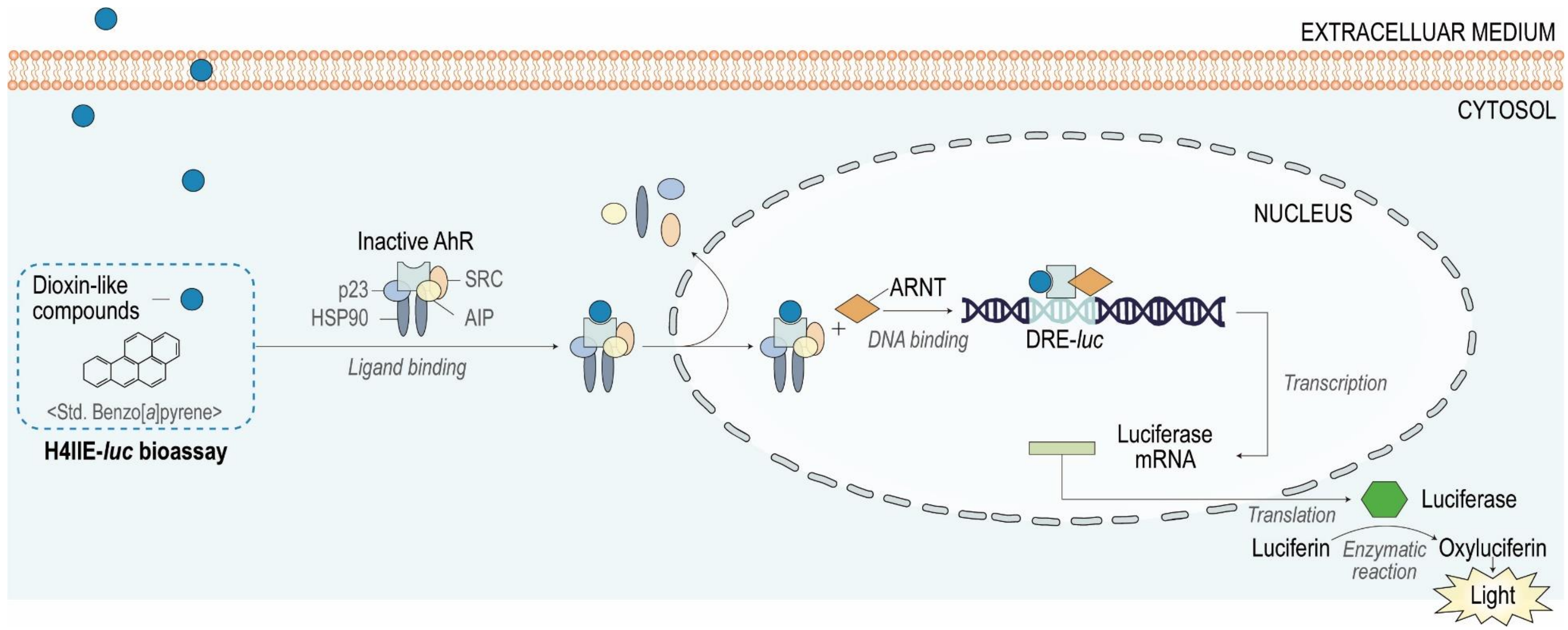

Figure S7. The molecular mechanisms of activation of gene expression by the aryl hydrocarbon receptor (AhR) mediated responses in cell. B[*a*]P; Benzo[*a*]pyrene; HSP90; 90 kDa heat shock protein, AIP AhR-interaction protein (also known as XAP2), ARNT; AhR nuclear translocator, DRE; dioxin responsive element.

Supplementary Tables

Table S1. Chemical compounds information of homologues of chrysene used in this study.

**Compounds**	**Abb.** [a)]	**Molecular formula**	**Number of benzene rings**	**Molecular weight**	**Log K_{ow}**	**CAS RN**	**Purity (%)**	**Vendor**
Chrysene	Chr	C18H12	4	228.294	5.81	218-01-9	> 98	Sigma-Aldrich (St. Louis, MO, USA)
Benzo[*a*]chrysene	BaC	C22H14	5	278.354	7.11	213-46-7	> 99	Tokyo Chemical Industry Ltd.
Benzo[*b*]chrysene	BbC	C22H14	5	278.354	7.11	214-17-5	> 98	Sigma-Aldrich (St. Louis, MO, USA)
Benzo[*c*]chrysene	BcC	C22H14	5	278.354	7.11	194-69-4	> 98	Sigma-Aldrich (St. Louis, MO, USA)
Dibenzo[*b*,*def*]chrysene	DbdC	C24H14	6	302.376	7.28	189-64-0	> 98	Tokyo Chemical Industry Ltd.
Dibenzo[*def*,*p*]chrysene	DdpC	C24H14	6	302.376	7.71	191-30-0	> 98	Sigma-Aldrich (St. Louis, MO, USA)
1-methylchrysene	1MC	C19H14	4	242.321	6.07 [b)]	3351-28-8	> 98	Sigma-Aldrich (St. Louis, MO, USA)
2-methylchrysene	2MC	C19H14	4	242.321	6.07 [b)]	3351-32-4	> 98	Sigma-Aldrich (St. Louis, MO, USA)
3-methylchrysene	3MC	C19H14	4	242.321	6.07 [b)]	3351-31-3	> 98	Sigma-Aldrich (St. Louis, MO, USA)
1-hydroxychrysene	1HC	C18H12O	4	244.287	5.04 [b)]	63019-38-5	> 98	Chiron
2-hydroxychrysene	2HC	C18H12O	4	244.287	5.04 [b)]	65945-06-4	> 98	Chiron
3-hydroxychrysene	3HC	C18H12O	4	244.287	5.04 [b)]	63019-39-6	> 98	Chiron

[a)] Abb.: Abbreviations; [b)] Estimated values.

Table S2. Physico-chemical properties of aryl hydrocarbon receptors.[3]

**Single letter code**	NFIFRTKHKLDFTPIGCDAKGQLILGYTEVELCTRGSGYQFIHAADILHCA ESHIRMIKTGESGMTVFRLLAKHSRWRWVQSNARLIYRNGRPDYIIATQR PLTDEE
**Number of residues**	107
**Molecular weight**	12381.1 g mol^{-1}
**Extinction coefficient**	16500 M^{-1} cm^{-1}
**Iso-electric point**	pH 9.14
**Net charge at pH 7.4**	4.1
**Estimated solubility**	Good water solubility
**Equation for net charge** [a)]	$\text{Net charge} = \sum_i N_i \frac{10^{pk_{a_i}}}{10^{pH} + 10^{pk_{a_i}}} - \sum_j N_j \frac{10^{pk_{a_i}}}{10^{pH} + 10^{pk_{a_j}}}$

[a)] N_i are the number, and pKa_i are the pKa values, of the N-terminus and the side chains of Arginine, Lysine, and Histidine.

Table S3. *In silico* toxicity prediction models estimating the potential toxicity of chrysene homologues.

Compounds	VEGA-QSAR [a)]	VitualToxlab [b)]	
	$1/LC_{50}$	AhR binding affinity (nM)	ToxPot
Chrysene	0.95	655	0.39
Benzo[*a*]chrysene	1.13	114	0.46
Benzo[*b*]chrysene	1.13	63.2	0.48
Benzo[*c*]chrysene	1.13	133	0.45
Dibenzo[*b*,*def*]chrysene	1.12	78.5	0.48
Dibenzo[*def*,*p*]chrysene	1.12	221	0.42
1-methylchrysene	0.53	593	0.39
2-methylchrysene	0.53	450	0.41
3-methylchrysene	0.53	501	0.40
1-hydroxychrysene	0.43	352	0.41
2-hydroxychrysene	0.43	466	0.41
3-hydroxychrysene	0.43	250	0.43

[a)] VEGA-QSAR estimated data (fish); [b)] potential toxicity and estimated affinity of binding to AhR.

Table S4. *In vitro* transactivation bioassay conditions for evaluating toxicities of chemical compounds.

**Cell line**	H4llE-*luc*
**ATCC#**	CRL-1548
**Cell type**	Recombinant (*luc*-gene)
**Mode of Action**	AhR-mediated potency
**Endpoint**	Luciferase activity
**Positive control**	Benzo[*a*]pyrene
**Culture condition**	37 °C, 5% CO2
**Exposure time**	4 h

References

(1) Waterhouse, A. *et al*. SWISS-MODEL: homology modelling of protein structures and complexes. *Nucleic. Acids. Res.* **46**, W296–W303 (2018).
(2) Wiederstein, M. & Sippl, M. J. ProSA-web: interactive web service for the recognition of errors in three-dimensional structures of proteins. *Nucleic Acids Res.* **35**, W407–W410 (2007)
(3) Lear, S. & Cobb, S. L. Pep-Calc. com: a set of web utilities for the calculation of peptide and peptoid properties and automatic mass spectral peak assignment. *J. Comput. Aided. Mol. Des.* **30**, 271–277 (2016).
(4) Shin, W.-H. *et al*. Prediction of protein structure and interaction by GALAXY protein modeling programs. *Bio. Design.* **2**, 1–11 (2014).
(5) Vedani, A. *et al*. OpenVirtualToxLab—a platform for generating and exchanging in silico toxicity data. *Toxicol. Lett.* **232**, 519–532 (2015).
